# The impact of ADHD symptoms and global impairment in childhood on working disability in mid-adulthood: a 28-year follow-up study using official disability pension records in a high-risk in-patient population

**DOI:** 10.1186/1471-244X-12-174

**Published:** 2012-10-19

**Authors:** Marianne Mordre, Berit Groholt, Berit Sandstad, Anne Margrethe Myhre

**Affiliations:** 1 Division of Mental Health and Addiction, Oslo University Hospital, P.O. Box 4959, Nydalen, 0424, Oslo, Norway; 2 Institute for Clinical Medicine, University of Oslo, Oslo, Norway; 3 Unit of Biostatistics and Epidemiology, Oslo University Hospital, Oslo, Norway; 4 Division of Mental Health and Addiction, Oslo University Hospital, Norway and Institute for Clinical Medicine, University of Oslo, Oslo, Norway

**Keywords:** ADHD, Conduct disorder, CGAS, Disability pension award

## Abstract

**Background:**

Individuals with ADHD have been associated with more employment difficulties in early adulthood than healthy community controls. To examine whether this association is attributable specifically to disturbance of activity and attention (ADHD) or to psychopathology in general, we wanted to extend existing research by comparing the rate of mid-adulthood working disabilities for individuals diagnosed with ADHD as children with the rate for clinical controls diagnosed with either conduct disorder, emotional disorder or mixed disorder of conduct and emotions.

**Methods:**

Former Norwegian child-psychiatric in-patients (n = 257) were followed up 17–39 years after hospitalization by record linkage to the Norwegian national registry of disability pension (DP) awards. Based on the hospital records, the patients were re-diagnosed according to ICD-10. Associations between the diagnoses, other baseline factors and subsequent DP were investigated using Kaplan–Meier survival analyses and logrank testing.

**Results:**

At follow-up, 19% of the participants had received a DP award. In the logrank testing, ADHD was the only disorder associated with a subsequent DP, with 30% being disabled at follow-up (p = 0.01). Low psychosocial functioning (assessed by the Children’s Global Assessment Scale) at admission uniquely predicted future DP (p = 0.04).

**Conclusions:**

ADHD in childhood was highly associated with later receiving a DP. Our finding of worse prognosis in ADHD compared with other internalizing and externalizing disorders in mid-adulthood supports the assumption of ADHD being specifically linked to working disability. Assessment of psychosocial functioning in addition to diagnostic features could enhance prediction of children who are most at risk of future disability.

## Background

Attention-deficit/hyperactivity disorder (ADHD) is generally recognized as a neurodevelopmental disorder with executive function deficits [1,2]. In numerous studies, ADHD symptoms have been found to be uniquely related to future academic difficulties, both in population-based studies [3-5], and in clinical follow-up studies [6-9]. However, in these studies, children have been followed only into adolescence and early adulthood [6-9]. This makes it difficult to conclude about the persisting disabling nature of ADHD symptoms. In general, there is a decline in ADHD symptoms into adulthood, but some symptoms (inattention) seem to persist [9,10]. To understand the long-term developmental course of ADHD, knowledge about vocational outcome in mid-life is of interest. Despite several community studies reporting work impairment in adults with ADHD [11-13], comparisons have been restricted to healthy non-psychiatric control groups, as in most of the clinical follow-up studies [6,9,14,15]. The use of healthy controls could make it difficult to determine the extent to which the poor outcome found at follow-up was likely to be a function of the severity of ADHD symptoms specifically, rather than a function of impairment related to severity of any mental disorder. This is of special importance in disorders that debut in childhood, as aspects of normal development may be suffering. There is considerable evidence that the long-term course of child-psychiatric disorders is associated with impaired functioning and continuity of symptoms into adult life [16-18].

The use of clinical controls to delineate outcome differences across childhood diagnoses could therefore be useful when examining different developmental trajectories. In general, externalizing disorders have been associated with a more negative outcome than internalizing disorders [16,19,20], with the worst outcomes being reported for co-morbid states of these disorders [20-22]. However, most of these studies have addressed the diagnostic course and criminality, and less is known about employment. Few studies have tested the validity of the persistent nature of ADHD by comparing working disabilities in individuals with ADHD with those who have either internalizing or other externalizing disorders.

In one study, Barkley et al. compared the outcomes of young adults with ADHD with individuals with internalizing disorders [23]. They found that overall impairment, including poor work performance, was higher in the ADHD group than in the internalizing group. In two community-based studies, children with ADHD symptoms were reported to fare worse in academic achievement than children with conduct symptoms [4,5]. These studies, however, were restricted to short-term follow-up comparisons of adolescents. Other studies have been limited by using controls that resembled ADHD groups [24,25], which made it difficult to draw conclusions about the specific nature of ADHD symptoms.

In sum, most studies are limited by follow-up periods that reach only into young adulthood and/or the lack of clinical control groups. Knowledge of the chronic course of work impairment into mid-adulthood relative to clinical controls is limited.

In the present study, we wanted to compare outcome differences into mid-adulthood between children with ADHD and those with either internalizing and/or other externalizing disorders in an in-patient population. Studying child-psychiatric in-patients with excessive symptom loads could delineate outcome differences among diagnostic groups. By following the individuals into their mid-thirties, and by obtaining information about disability pension (DP) awards for these individuals, we could provide a comprehensive picture of occupational disability in adults with a childhood history of severe mental disorders. We wanted to see if working disability was associated specifically with ADHD, or whether it was an experience shared with other disorders diagnosed in childhood.

We hypothesized that former child-psychiatric in-patients with ADHD would have a higher rate of working disability relative to other clinical groups. We also hypothesized that individuals with co-morbid ADHD and conduct disorder would be more disabled than individuals with co-morbid conduct disorder and emotional disorder, as we assumed that working disability is specifically associated with ADHD.

As a diagnosis is only one component of the overall assessment process, and because symptoms and impairment are partly distinct dimensions that do not always correlate [7,26-28], a final issue was to examine whether the global assessment of psychosocial functioning in childhood could enhance outcome predictions.

## Methods

### Study population

The study population consisted of 258 child-psychiatric in-patients with intellectual level within the normal range. They were part of a larger study population consisting of all consecutively admitted in-patients (n = 550) at the children’s unit at the National Centre for Child and Adolescent Psychiatry in Oslo (NCCAP), Norway, from January 1968 to October 1988. The complete study population has been described in detail elsewhere [29,30]. The children were admitted as in-patients because of their long lasting and complex problems. They were referred to the hospital from all over the country for assessment and in-patient treatment when local out-patient clinics were short of professional expertise. The children’s unit at NCCAP provided specialized treatment for children up to 13 years. In the present study, individuals with emotional disorders, mixed disorder of conduct and emotions, conduct disorder and/or hyperkinetic disorders were included. Individuals with other psychiatric disorders and/or mental retardation (n = 292) were excluded. One person had emigrated before the age of 16 years, which was the youngest age for registration of a DP award in Norway during the study period, and was consequently excluded from the study. Thus, 257 participants were included in this study.

The gender distribution in the group was 82 (32%) girls and 175 (68%) boys. The mean age at admission was 8.7 years (SD 2. 3, range 2–13), with no gender differences. Fifty-six per cent of the patients were admitted to the family ward, 40% to the in-patients long-term ward and 4% to the day care ward. The mean length of stay was 1.1 months (SD 0.3, range 1–3) at the family ward, 8.2 months (SD 8.8, range 0.5-32) at the in-patients long-term ward and 24.4 months (SD 17.8, range 6–50) at the day-care ward. In total, 17% of the patients were admitted more than once. The mean age at follow-up (when those who had emigrated or died were excluded) was 36.9 years (SD 7.0, range 21–50), with a mean follow-up period of 28.2 years (SD 6.8, range 17–39). A total of 11 (4%) participants had died and four (2%) had emigrated.

### Outcome variables

At follow-up in December 2005, the participants were identified by personal identification number in the population register at Statistics Norway. By record linkage, information about the DP award was obtained from Statistics Norway’s events database FD–Trygd, which includes information from the Norwegian Labour and Welfare Organization. All citizens of Norway are eligible after 18 years of age (previously 16 years, until January 1^st^ 1998) to be granted DP for an acknowledged medical condition that causes >50% lasting reduction in work capacity. Entitlement to a DP is not means tested; it is solely a public responsibility.

The Norwegian Labour and Welfare Organization records information on all DP awards. Correct registration is a prerequisite for transfers of payment, and the records are thus highly accurate.

### Measures

#### Mental health (ICD-10)

Based on all clinical information in the comprehensive hospital records, all the 257 patients were re-diagnosed according to the ICD-10 [31]. The diagnosis of greatest clinical importance (principal diagnosis) was pre-empted (Table 1). All the diagnoses were based on a consensus of two or more clinicians. (The diagnostic procedure has been described in more details in a previous report [29]).

**Table 1:** Distribution and descriptive characteristics of diagnostic groups at admission

**Diagnostic groups**	**CD**	**ADHD**	**ED**	**MCE**	**Total study population**	ANOVA with post hoc*
N	39	53	98	67	257	
CGAS	44.0(6.5)	41.9(5.5)	48.9(9.6)	43.4(6.5)	45.3(8.1)	CD=^b^ADHD=MCE<ED, p<0.05
Age at admission	8.9(1.7)	8.0(2.3)	8.9(2.7)	8.8(1.9)	8.7(2.3)	P= 0.13
Male gender^a^	31(80)	44(83)	52(53)	48(72)	175(68)	ED< ADHD=CD=MCE, p<0.05
CFD	4.9(1.0)	4.0(1.4)	4.1(1.3)	4.7(1.1)	4.4(1.3)	ED=ADHD<CD=MCE, p<0.05

ANOVA with post hoc analysis to provide differences between diagnostic groups.

*ANOVA post hoc if p<0.05 in one-way Anova.

^a^ Pearson’s chi square/Fisher’s exact test.

^b^ non-significant differences.

*CD*, Conduct disorder; *ADHD*, Hyperkinetic disorder; *ED*, Emotional Disorder; *MCE*, Mixed disorder of conduct and emotions; *CGAS*, Children’s Global Assessment Scale; *CFD*, Chronic family difficulties scale.

#### Children’s Global assessment scale (CGAS)

The children were reassessed on the CGAS, a global assessment of the child’s psychosocial functioning [32], according to how they were described in the hospital records at the time of admission (Table 1). The CGAS is a widely used clinician-rated scale that assigns a single summary score from 1 to 100, with 1 indicating the most severely disordered child and 100 the best-functioning child [32,33]. Anchors at 10-point intervals include descriptors of functioning for each interval. The instrument has been validated against many different psychiatric assessment scales [34], and it is good at distinguishing cases from non-cases [35,36].

#### Level of cognitive abilities

An assessment of each participant’s cognitive level was based on clinical findings and psychometric and pedagogic test results reported in the hospital records. For children of school age, teachers at NCCAP’s affiliated school performed systematic pedagogic evaluations.

Diagnostic criteria for mental retardation were used according to the ICD-10. Only those patients with intellectual level in the normal range were included, as it was important to isolate the confounding effect of mental retardation when comparing diagnostic groups.

#### Socio-demographic variables

Gender was registered at baseline and reported in Table 1. Based on all the information available in the hospital records of the past and present family situation, we applied a global assessment of chronic family difficulties (CFD) [37]. Socio-economic conditions, social network, marital or family discord and current/previous physical and mental health of the family members were recorded. The total burden of difficulties was scored on an interval scale from 0 to 6, with 0 indicating no sign of chronic family difficulties and a score of 6 indicating severe difficulties/very disturbed family environment (Table 1).

#### Inter-rater study

An inter-rater reliability study was carried out for the ICD-10 diagnoses, CGAS, CFD and cognitive level, and solid reliabilities were found [29].

### Statistical methods

Categorical variables were examined using Pearson’s chi-square test and Fisher’s exact test, as appropriate. Continuous variables were examined using Student’s two-sample *t-*test. A one–way ANOVA with a post-hoc comparison (Tukey) was conducted to explore differences in age, CFD and CGAS between diagnostic groups.

Possible associations between diagnostic groups and other characteristics of the children and a subsequent DP were tested using univariate survival analyses according to the Kaplan-Meier method and logrank testing. Using stratified logrank analyses, we examined the association between diagnosis and subsequent DP, adjusting for CGAS, CFD and gender.

In the survival analyses, participants were followed from the age of 16 years, which was the youngest age for registration of a DP during the study period, until a DP award was first registered, or otherwise, until their date of emigration, death, or follow-up in December 2005 for those who did not receive a DP. The study group was compared with the average Norwegian population in 2005 by using tables statistics [38]. SPSS version 18 was used for the statistical analyses.

### Ethics

The study was approved by the Regional Committee of Ethics in Medical Research, the Department of Health and Social Services, and the Norwegian Data Inspectorate.

## Results

### ICD-10 diagnostic groups

Conduct disorder (CD) (F91) was diagnosed in 39 children (15%) according to the ICD-10. Hyperkinetic disorder (ADHD) (F90) was diagnosed in 53 individuals (21%), corresponding to the ADHD of combined type according to the DSM-IV criteria [39], except for three participants who fulfilled only the criteria for ADD. Thirty-two of these children (60%) also met the criteria for conduct disorder (F90.1). Emotional disorder (ED) (including emotional disorders in childhood (F93), anxiety and other neurotic disorders (F40–F49), mood disorders (F30–F39), eating disorders (F50) and mutism (F94.0) was diagnosed in 98 children (38%) according to the ICD-10. Mixed disorder of conduct and emotions (MCE) (F92) was diagnosed in 67 children (26%) who met the criteria for both an emotional disorder and a conduct disorder.

Characteristics of the diagnostic groups are given in Table 1. With the exception of the emotional group, which had a greater percentage of females and higher CGAS scores at admission, there were no significant differences between diagnostic groups with regard to age, gender and CGAS score at admission.

### Disability pension award

At follow-up, 49 persons (19%) had received a DP award, with no significant gender differences (Table 2). The disability rate was markedly higher than the rate for the same age groups in the general population, where a crude estimate was 5% [40]. The mean age when the DP was awarded was 22.8 years (SD 8.4, range 16–42) at the end of the follow-up.

**Table 2:** Vulnerability factors for disability pension award

**Vulnerability factors**	**N= 257**	**DP**	**Non-DP**	**P-value**
		**N=49**	**N= 208**	**Log- rank test**
		**N (%)/Mean (SD)**	**N (%)/Mean (SD)**	
**Mental health (ICD-10)**				
CD	39	4(10)	35(90)	0.11
ADHD	53	16(30)	37(70)	**0.01**
ADHD only	21	5(24)	16(76)	
Co-morbid ADHD and CD	32	11(34)	21(66)	
ED	98	15(15)	83(85)	0.20
MCE	67	14(21)	53(79)	0.61
**CGAS**				**0.04**^**a**^
CGAS <40	51	16(31)	35(69)	
CGAS 40–49	139	24(17)	115(83)	
CGAS ≥50	67	9(13)	58(87)	
**Sociodemographic variables**				
Male gender	175	34(19)	141(81)	0.72
Female gender	82	15(18)	67(82)	
CFD	256	4.5(1.2)	4.3(1.3)	0. 91

Difference in prevalence of psychiatric disorders and other vulnerability factors between subjects who received disability pension (DP, N=49) and who did not receive DP (Non-DP, N=208) during the follow-up period. Significant p-values of the logrank testing are given in bold.

^a^ Logrank test with linear trend for factor levels.

*CD*, Conduct disorder; *ADHD*, Hyperkinetic disorder; *ED*, Emotional Disorder; *MCE*, Mixed disorder of conduct and emotions; *CGAS*, Children’s Global Assessment Scale; *CFD*, Chronic family difficulties scale.

### Vulnerability factors of the disability pension award

**Figure 1:**
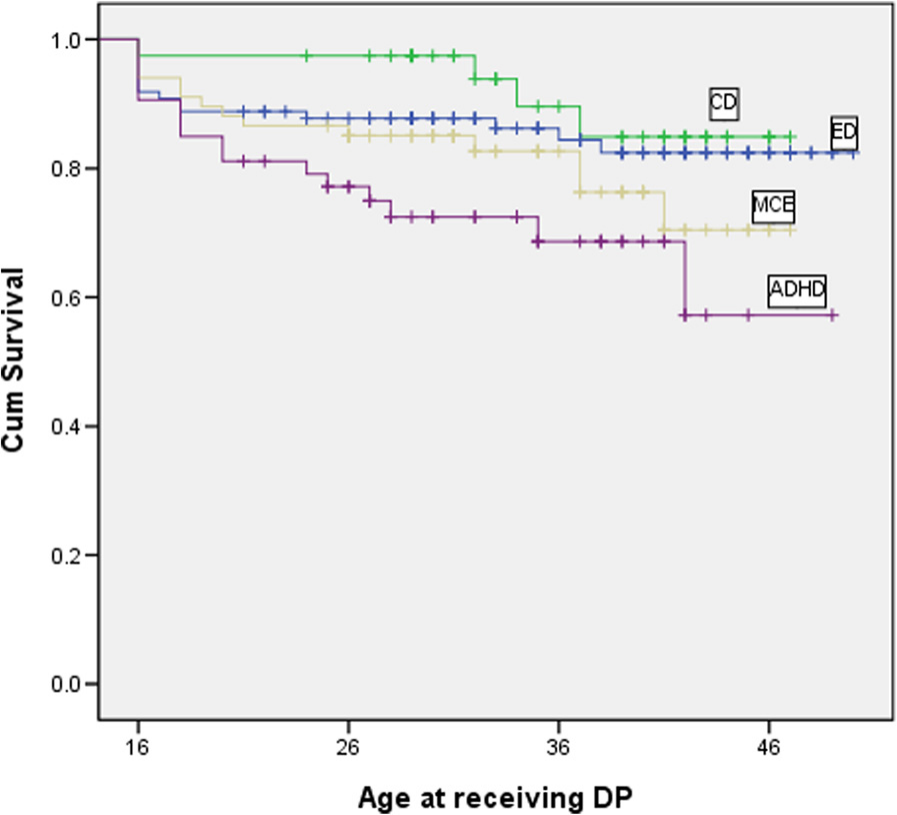
**Kaplan-Meier curves depicting the age at receiving disability pension (DP) in patients with different ICD-10 diagnoses at admission.** CD, Conduct disorder; ED, Emotional disorder; MCE, Mixed disorder of conduct and emotions; ADHD, Hyperkinetic disorder.

**Figure 2:**
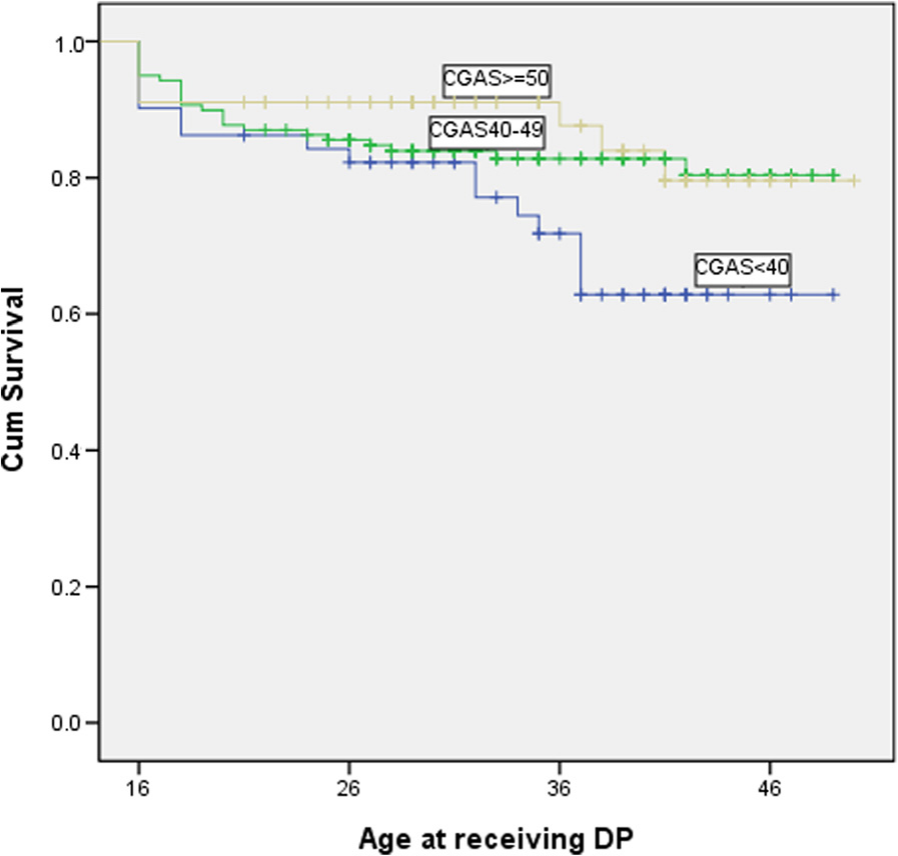
Kaplan-Meier curves depicting the age at receiving disability pension (DP) in patients with different Children’s global assessment scale (CGAS) scores at admission.

The prevalence of DP awards and the p-value for the logrank test of diagnostic groups and other vulnerability factors are summarized in Table 2. Figures 1 and 2 show the Kaplan-Meier plots for variables most strongly related to receiving the DP award.

We made an overall diagnosis variable consisting of the four diagnostic categories (CD, ADHD, MCE and ED) to be used in the Kaplan-Meier and logrank analyses (Figure 1, Table 2). The diagnosis variable was significantly associated with a subsequent DP award (p = 0.03). We also made one categorical variable for each diagnostic category: ADHD was the only disorder that was significantly associated with a subsequent DP, with 30% of these patients being disabled at follow-up (p = 0.01) (Table 2). When we split the ADHD group into those with ADHD only and those with ADHD and a co-morbid conduct disorder, there were not enough patients in each group to predict DP awards separately for the two groups. The higher disability rate in the group with co-morbid conduct disorder did not differ significantly from the rate for the ADHD-only group (34% vs. 24%, p = 0.54, by Fischer’s exact test). Our results thus suggest that co-morbid conduct disorder seemed to have no more than an additive effect. Similarly, there seemed to be no more than an additive effect for those with both emotional disorder and conduct disorder (MCE) relative to those who had either disorder alone (15% vs. 21%, p = 0.35, and 10% vs. 21%, p = 0.19, by Pearson chi-square).

The CGAS score was condensed to a three-level score to be used in the Kaplan-Meier plot and in the logrank test with linear trends for factor levels (Figure 2, Table 2). The CGAS score at admission was significantly associated with a DP award at follow-up (p = 0. 04). Gender and CFD were not associated with a DP award.

To adjust for the different CGAS scores between diagnostic groups, we ran stratified logrank analyses in which the diagnostic groups were stratified by the CGAS three-level score. Hyperkinetic disorder was still the only diagnosis associated with a DP award (data not shown). We also ran logrank analyses in which the diagnostic groups were stratified by gender and CFD. The results did not change (data not shown). Lastly, the relationship between CGAS and DP remained similar when the analysis was stratified by diagnostic groups (data not shown).

## Discussion

In this study, we compared the disability outcomes of ADHD patients with outcomes for patients with other externalizing and internalizing disorders. In line with our hypotheses, ADHD in childhood was highly associated with working disability in mid-adulthood, with the disability rate being highest when there was a co-morbid conduct disorder. We also found that individuals with co-morbid ADHD and conduct disorder were more disabled than were those with co-morbid emotional and conduct disorder, which reinforces the assumption that working disability is specifically associated with ADHD symptoms. Furthermore, assessment of psychosocial functioning at admission provided unique information about children at risk of a poor outcome.

### Disability pension award

At the end of a 28-years study period, 19% of the individuals in this study had received a DP award, a markedly higher rate than that for the general population. As a DP award is most often a permanent benefit, awarded when treatment and extensive rehabilitation have failed, it is indicative of the severity of the work impairment these individuals have.

Our finding is in line with those from previous studies, which have shown that individuals with child and adolescent psychiatric disorders have disability rates ranging from 13% to 50% at follow-up [41-46]. Because mental retardation, organic disorders and pervasive developmental disorders are well-known predictors of DP [30,45,47], individuals with such disturbances were excluded in the present study. This probably explains the somewhat lower DP rate in our population relative to the 50% DP rate found in two similar studies of adolescent in-patients, in which no such exclusions were performed [42,45]. However, all these studies present strong evidence for the relationship between severe psychiatric disorders in childhood and severe work impairment in adulthood.

### Vulnerability factors of the disability pension award

#### ICD-10 disorders

ADHD was the only disorder significantly associated with a subsequent DP award in the present study. The disability rate in the ADHD group was about twice that of groups with CD or ED, which suggests that work impairment is related to specific symptoms central to ADHD. Our finding thus extends previous research reporting this elevated risk for ADHD probands relative to non-clinical controls [11-13,48,49].

In two recent Norwegian studies of adult out-patients with ADHD, early intervention was an important predictor of being employed in adulthood [48,50]. Even though all of our patients received early intervention during hospitalization, their disability rate was markedly elevated and was as high as in these previous studies. However, our children were severely affected in-patients, and their poor outcome was probably influenced by their symptom severity. More than half of the children (60%) had a co-morbid conduct disorder, and the prognosis for such individuals has been consistently reported to be even worse than for individuals with ADHD alone [24,51,52].

If we take the co-morbidity into account, it could perhaps be argued that the high rate of DP at follow-up, was a function of CD rather than childhood ADHD. However, CD alone or combined with ED was not associated with a DP award within this study population, and the DP rate was more than twice as high in the ADHD only group relative to the rate in the CD group. Thus, it seems that ADHD was the main contributor of the high DP rate found at follow-up, and that co-morbid CD had no more than an additive effect.

The lack of association between CD and DP is consistent with findings in previous follow-up studies of in-patients [45,47]. Similarly, a population-based study reported no association between employment difficulties and conduct problems when attention problems were adjusted for [53]. This gives support to the assumption of different aetiologies in ADHD and CD, underlying the neuropsychological characteristics of ADHD [2]. However, childhood CD has been consistently associated with delinquency [29,54-57], and many of these patients may have spent periods of their lives in prison. Consequently, the need for alternative financial support may have been reduced and/or these individuals’ poor functioning has not been ascribed to disorders that give entitlement to a DP award. Worth noting, our individuals with CD had elevated DP rate relative to the rate in the general population. Previous research in less severe populations of out-patients and community samples has shown that early-onset conduct problems constitute a risk for a wide range of later adverse outcomes, which include mental health problems [58] and unemployment [59-61]. Considering all the serious implication of adult life in children with conduct problems, early intervention should be stressed to mitigate the poor prognosis.

In the present study, the combination of conduct disorder and emotional disorder appeared to be more disabling than either disorder alone. Our findings are thus consistent with previous research showing that individuals with co-morbid disorders have worse outcomes than those without [20,22]. Given the increased impairment in children with co-morbid disorders, accurate diagnosis and monitoring of co-morbidity are critical.

In line with our hypothesis, we found that individuals with combined ADHD and conduct disorder had a higher rate of DP than did those with combined emotional and conduct disorder, giving further support to the assumption that working disability is to some extend specifically associated with ADHD. The poor academic achievement reported for ADHD individuals may lead to low grade occupations [62,63]. DP has been found to be independently associated with low educational level [64]. Intensified treatment strategies that optimize school functioning could probably have significant contribution to promote educational attainment and later occupational functioning in this group.

#### Children’s Global assessment scale (CGAS)

In this study, psychosocial impairment (assessed by CGAS score) was an important vulnerability factor for a DP award, even when adjusting for the presence of an ICD-10 disorder. Our finding illustrates the importance of evaluating both symptoms and functional impairment as part of a diagnostic assessment when studying an individual’s developmental course. Attempts to link impairment to specific diagnoses have turned out to be difficult [27], and severity of symptoms and level of functioning have been claimed to be partly independent dimensions that do not always correlate [26,28]. Although the CGAS is problematic because symptoms and psychosocial impairment are conflated in a single scale score, our findings suggest that this global measure can identify individuals at risk of continuing problems decades later. Because the CGAS total score is correlated with an individual’s overall psychosocial adjustment, it may promote more accurate prognoses than those assessments based on diagnostic features only.

#### Socio-demographic variables (gender/CFD)

In line with previous clinical research [45,47], neither gender nor family difficulties (assessed by the chronic family difficulties scale, CFD) were associated with a subsequent DP award. Our failure to find an association between CFD and DP could be an artefact, however, because substantial family difficulties were reported for all of our children. In two recent Nordic population-based studies, unfavourable conditions in childhood, including family difficulties, were significantly associated with subsequent DP [65,66]. Likewise, in a four-year follow-up study of 140 ADHD children and 120 community controls, Biederman et al. found that family adversities were highly associated with a poor outcome (persistence of ADHD) [67]. We need further large-scale intervention studies to determine whether targeting family difficulties is likely to reduce the association between child-psychiatric patients and future DP.

### Strengths and limitations

In this study, data were collected over a period varying from 17 to 39 years in a longitudinal follow-up study to examine the link between psychiatric disorders in childhood and a later DP award. The study’s strengths are the long follow-up period and the relatively large number of patients included. It is a nationwide study, in which there should be no regional admission bias. Because of the egalitarian Norwegian society where all citizens are entitled the same health care system, the socio-economic position of the family was not the predominating factor when these patients were hospitalized.

The scoring was performed blind to outcome, as re-diagnosis and scoring of the study population were completed before the outcome information was collected. The outcome variable, DP, is based on a high-quality national register.

This study has several limitations, however. All information was based on chart reviews from hospital records, which are not always reliable scientific sources. However, the hospital records were of good quality, giving a detailed and thorough description of the patients’ symptoms, psychometric test results and family adversities. Experienced psychiatrists completed the re-diagnosis and scoring of the data from the study sample, and they ensured that consensus-based best-estimate diagnoses were made as accurately as possible according to the current ICD-10. Inter-rater reliability was high [29], in line with previous research, for which the validity of file-based diagnostic ratings has been found satisfactory [68,69].

We have no estimate of the persistence of symptoms into adulthood in our children, but previous research has shown that symptom persistence is associated with symptom severity and co-morbidity in childhood [67,70,71]. Furthermore, adult disorders are often preceded by their juvenile counterparts [17]. However, because there were only two time points (i.e., childhood diagnoses and adult disability records) in the present study, we are restricted to reporting differences in prognosis across childhood diagnoses.

The DP award was the outcome measure in the present study, and this measure can never be sufficient to judge broader outcomes for individuals. However, the DP is an indicator of impairment in one major life activity that provides important information about adult outcomes.

Our group of individuals who were awarded the DP at follow-up provided insufficient statistical power to predict DP in single and co-morbid states of ADHD. Replication with larger groups is needed to predict DP in these subgroups separately.

Because we only had three individuals with ADD, we could not predict DP in the inattentive group relative to those with hyperactivity.

The factors identified in this study are not causative, but they should be considered as possible vulnerability factors that increase the risk of receiving a DP in an in-patient population. It is important to realize that this population consisted of severe cases that might have worsened the long-term outcome, and the results cannot immediately be generalized to out-patients. As criteria for obtaining DP vary considerably between countries, generalization of the findings is limited to nations with similar welfare systems.

## Conclusions

In this longitudinal study of child-psychiatric in-patients, we found that working ability was severely affected in mid-adulthood. ADHD appeared to be specifically linked to working disability compared with other externalizing and internalizing disorders. Considering the early onset and often chronic course of ADHD, direct primary prevention programs in preschools and schools should be endeavoured to mitigate academic underachievement. Given the increased impairment in children with co-morbid conduct disorder, early targeting of conduct problems should be emphasized. Regardless of diagnostic features, the assessment of psychosocial functioning could improve identifying children being most at risk of future disability. The chronic course and high rate of disability in child-psychiatric in-patients illuminate the importance of improving early intervention among such patients. Further research is highly needed to identify factors preventing long-term disability.

## Abbreviations

MM: Marianne Mordre; BG: Berit Groholt; BS: Berit Sandstad; AMM: Anne Margrethe Myhre.

## Competing interests

The authors declare that they have no competing interests.

## Authors’ contributions

All authors (except BS) conceived of and designed the study. MM participated in the collection of data, performed statistical analyses and drafted the first manuscript. BG participated in the collection of data, helped with statistical analyses and made significant contribution to the final draft. BS made significant contribution to the statistically analyses and critically reviewed the manuscript. AMM participated in the collection of data, made significant contribution to the final draft and supervised the work and critically reviewed the manuscript. All authors read and approved the final manuscript.

## Pre-publication history

The pre-publication history for this paper can be accessed here:

http://www.biomedcentral.com/1471-244X/12/174/prepub
